# Ethnicity and sexual risk in heterosexual people attending sexual health clinics in England: a cross-sectional, self-administered questionnaire study

**DOI:** 10.1136/sextrans-2017-053308

**Published:** 2018-03-08

**Authors:** Rachel Margaret Coyle, Ada Rose Miltz, Fiona C Lampe, Janey Sewell, Andrew N Phillips, Andrew Speakman, Jyoti Dhar, Lorraine Sherr, S Tariq Sadiq, Stephen Taylor, Daniel R Ivens, Simon Collins, Jonathan Elford, Jane Anderson, Alison Rodger

**Affiliations:** 1 Research Department of Infection and Population Health, University College London, Royal Free Hospital, London, UK; 2 Staffordshire and Stoke on Trent Partnership NHS Trust, Leicester, UK; 3 Institute for Infection and Immunity, St George’s, University of London, London, UK; 4 Birmingham Heartlands Hospital, Heart of England NHS Foundation Trust, Birmingham, UK; 5 Marlborough Department of Sexual Health, Royal Free Hospital, London, UK; 6 HIV i-Base, London, UK; 7 School of Health Sciences, City University London, London, UK; 8 Centre for the Study of Sexual Health and HIV, Homerton University Hospital NHS Foundation Trust, London, UK

**Keywords:** ethnic groups, sexual behaviour, sexual networks, sexually transmitted diseases

## Abstract

**Objectives:**

In the UK, people of black ethnicity experience a disproportionate burden of HIV and STI. We aimed to assess the association of ethnicity with sexual behaviour and sexual health among women and heterosexual men attending genitourinary medicine (GUM) clinics in England.

**Methods:**

The Attitudes to and Understanding of Risk of Acquisition of HIV is a cross-sectional, self-administered questionnaire study of HIV negative people recruited from 20 GUM clinics in England, 2013–2014. Modified Poisson regression with robust SEs was used to calculate adjusted prevalence ratios (aPR) for the association between ethnicity and various sexual risk behaviours, adjusted for age, study region, education and relationship status.

**Results:**

Questionnaires were completed by 1146 individuals, 676 women and 470 heterosexual men. Ethnicity was recorded for 1131 (98.8%) participants: 550 (48.6%) black/mixed African, 168 (14.9%) black/mixed Caribbean, 308 (27.2%) white ethnic groups, 105 (9.3%) other ethnicity. Compared with women from white ethnic groups, black/mixed African women were less likely to report condomless sex with a non-regular partner (aPR (95% CI) 0.67 (0.51 to 0.88)), black/mixed African and black/mixed Caribbean women were less likely to report two or more new partners (0.42 (0.32 to 0.55) and 0.44 (0.29 to 0.65), respectively), and black/mixed Caribbean women were more likely to report an STI diagnosis (1.56 (1.00 to 2.42)). Compared with men from white ethnic groups, black/mixed Caribbean men were more likely to report an STI diagnosis (1.91 (1.20 to 3.04)), but did not report risk behaviours more frequently. Men and women of black/mixed Caribbean ethnicity remained more likely to report STI history after adjustment for sexual risk behaviours.

**Discussion:**

Risk behaviours were reported less frequently by women of black ethnicity; however, history of STI was more prevalent among black/mixed Caribbean women. In black/mixed Caribbean men, higher STI history was not explained by ethnic variation in reported risk behaviours. The association between STI and black/mixed Caribbean ethnicity remained after adjustment for risk behaviours.

## Introduction

Sexual health outcomes vary by ethnicity, a disparity recognised in the UK and globally.^1–3^ Despite making up 1.8% of the UK population, black African men and women account for almost one-third of people accessing HIV care.^4^ In 2013, Public Health England (PHE) estimated that approximately 57% of HIV infections among heterosexual individuals were likely to have been acquired in the UK,^5^ highlighting the need for prevention strategies. People of black ethnicity also account for a disproportionate number of UK STI diagnoses.^1^ A recent study of STI diagnosis in UK genitourinary medicine (GUM) clinics found that the adjusted incidence rate ratio for gonorrhoea in black Caribbean men and women was almost six times that of white British individuals.^6^

A number of studies have explored the association between ethnicity, sexual behaviour and sexual health in the UK. More than 1300 black African men and women participated in the 2007 Mayisha II study, 76% of whom provided an anonymised sample for HIV testing.^7^ HIV prevalence in Mayisha II was significantly associated with history of STI and reporting two or more new partners in the previous year.^7^ Natsal-2 (data collection 1999–2001) reported that compared with white British men, men of black ethnicity were more likely to report sexual risk behaviour.^8^ Similar findings have been demonstrated in further UK studies.^2 9 10^ Jayakody *et al* found that among adolescent men reporting multiple risk behaviours was similar across white British, black African and black Caribbean young men, and black Caribbean men were more likely to report condom use.^10^

The over-representation of black ethnicity individuals in STI statistics in the UK has been consistent over more than a decade.^2 8 9^ Moreover, studies often lack sufficient statistical power to analyse separately individuals of different black ethnicities.^1 11 12^ The Patient Access and the Transmission of Sexually Transmitted Infections study demonstrated evidence that sexual behaviours and outcomes differ between black ethnic groups^13^ and this has been^13^ highlighted by PHE as a key area for research.^1^ The Attitudes to and Understanding of Risk of Acquisition of HIV (AURAH) study is a cross-sectional questionnaire study which collected data on sociodemographic characteristics, health, lifestyle and sexual behaviours in individuals without diagnosed HIV attending GUM clinics in England.^14^ This analysis aims to describe the relationship between sexual behaviour, sexual health and ethnicity in individuals attending GUM clinics in the UK.

## Methods

The AURAH study methods have been described in detail elsewhere.^14^ Briefly, AURAH was a cross-sectional, self-administered questionnaire study with participants recruited from 20 GUM clinics across England between June 2013 and November 2014. Inclusion criteria were: not diagnosed with HIV at time of recruitment, aged 18 years or over and attending for routine STI and/or HIV testing. Participants diagnosed with HIV at the clinic visit were retained in the sample (n=9). Initially, study recruitment was unrestricted. After 6 months, recruitment was targeted at men who have sex with men (MSM) and individuals of black ethnicity.

The AURAH questionnaire included questions on sociodemographic characteristics, physical and mental health, attitudes to HIV, use and knowledge of post-exposure prophylaxis and pre-exposure prophylaxis, sexual activity, lifestyle and HIV testing preferences. Participants were asked if they were in an ongoing relationship with a partner (wife, husband, civil partner, boyfriend, girlfriend).

The following sexual risk behaviour and sexual history measures are used: recent (within 3 months) condomless vaginal or anal sex—categorised as no condomless sex, condomless sex with a long-term partner only, or condomless sex with a non-regular partner/s; recent condomless sex with two or more partners; recent condomless sex with a partner of unknown or positive HIV status; having two or more new sexual partners in the last year; self-reported history of STI diagnosis in the last year (excluding STI diagnosed at recruitment); low self-efficacy in relation to condom use—participants were deemed to demonstrate low self-efficacy if they disagreed/strongly disagreed with the statement ‘I feel confident that, if I want to, I can make sure a condom is used during sex with any partner, in any situation’, or agreed/strongly agreed with the statement ‘I find it difficult to discuss condom use with any new sexual partner’. Participants were also asked if they had a history of HIV testing.

For sexual behaviour measures, missing data were taken to indicate absence of the behaviour; a sensitivity analysis excluding missing cases was also undertaken.

Sociodemographic variables included: study region, UK birth, employment, housing, financial hardship (reporting insufficient money to pay for basic needs) and education level. Alcohol intake was assessed using the first two questions of the Alcohol Use Disorders Identificaton Test (AUDITC)_ tool (≥6 is used to indicate high-risk alcohol use).^15^ Recreational drug use in the past 3 months was recorded.

### Ethnicity

Participants selected their ethnic group from a list based on the UK census. Individuals reporting black or mixed African ethnicity were categorised as black/mixed African; those reporting black or mixed Caribbean ethnicity were categorised as black/mixed Caribbean. All white ethnic groups were categorised as white ethnicity, individuals from all other ethnic groups were categorised as ‘other ethnic group’. Participants in whom ethnicity was missing (n=15) were excluded. A sensitivity analysis was carried out with ethnic groups recategorised as: black African only, black Caribbean only, white ethnic groups and all other ethnic groups including individuals reporting mixed ethnicity.

### Sample size

The AURAH study aimed to recruit 2000 individuals, of whom 1000 would be MSM and 1000 would be heterosexual men and women including 600 black African men and women. This sample size was intended to allow for calculation of the proportion of individuals reporting condomless sex with a partner of unknown or positive HIV status. For the planned sample size of approximately 500 black African women and 300 black African men, prevalences of 5% would be estimated with 95% CIs of ±1.9% and ±2.5%, respectively.^14^ The relationship between ethnicity and sexual health was addressed as a secondary analysis of the study data.

### Statistical analysis

Analysis was performed separately for men and women. Bisexual women were retained in, and gay women were excluded from, the analysis of sexual behaviour.

Χ^2^ tests were used to assess the association between ethnicity, sexual behaviour and sexual history. Modified Poisson regression with robust error variances was used to calculate adjusted prevalence ratios and 95% CIs for the association of ethnicity and sexual behaviour and history. This is an accepted alternative to logistic regression in the analysis of binary outcomes, the main advantage of which is that it estimates the readily interpretable prevalence ratio rather than the OR.^16^ The first adjusted model included ethnicity, age, study region, education and relationship status, the second adjusted models included additional adjustment for alcohol and drug use. The effect of adjustment for sexual behaviour measures on the association between ethnicity and STI history was assessed.

Analyses were performed using Stata V.14.1.

## Results

**Figure 1:**
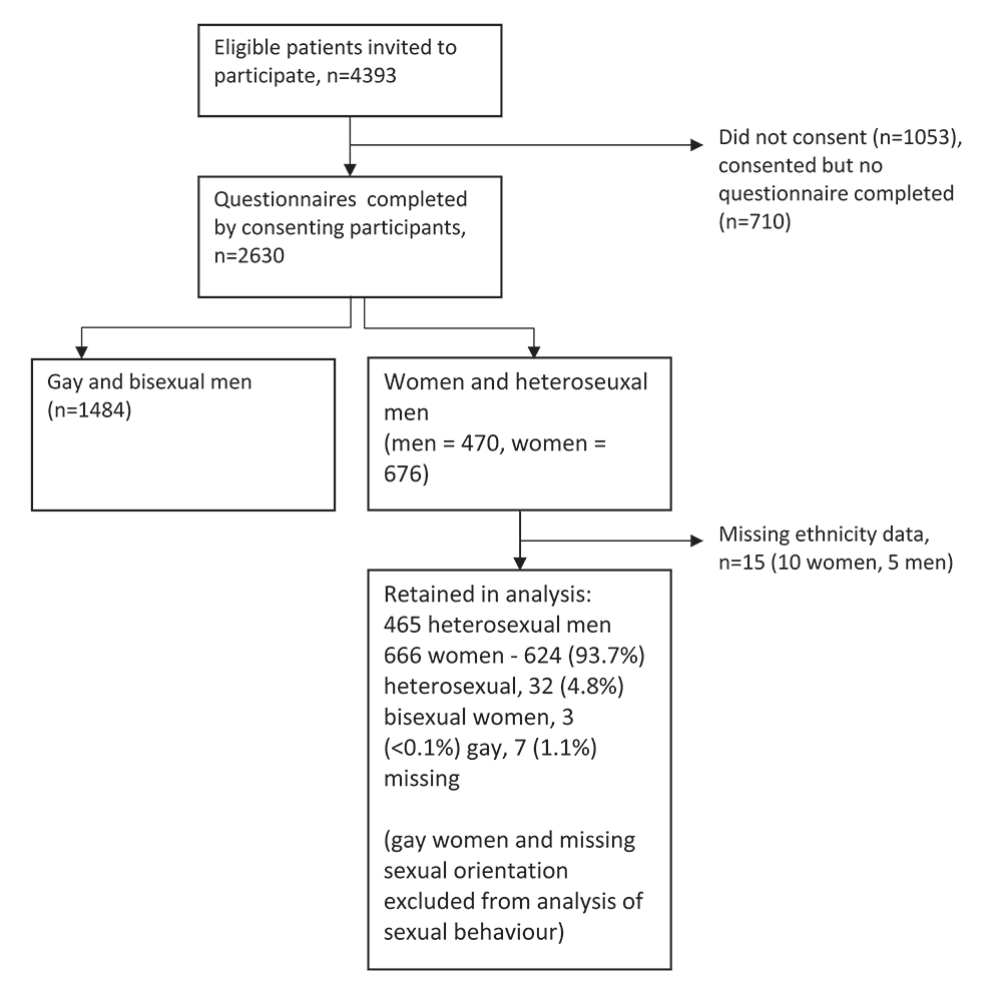
Recruitment, inclusions and exclusions.

There were 2630 participants in the AURAH study (response rate 60%). Recruitment is summarised in figure 1. Following exclusion of men who identified as gay or bisexual and transgender individuals reporting sex between biological men, there were 470 (41.0%) heterosexual men and 676 (59.0%) women. Data on sexual orientation were available for 1129 of 1146 individuals (98.5%). All 465 men included in the analysis were heterosexual. Of the 666 women with complete ethnicity data, 624 (93.7%) were heterosexual, 32 (4.8%) were bisexual and 3 (<0.1%) were gay (7 missing, <0.1%).

Ethnicity was recorded for 1131 (98.7%) participants. There were 550 (48.6%) black/mixed African participants, 168 (14.9%) black/mixed Caribbean participants, 308 (27.2%) participants from white ethnic groups and 105 (9.3%) other ethnicity participants.

There was significant variation in the sociodemographic characteristics of participants of different ethnicities (table 1). Compared with black/mixed Caribbean and white women, black/mixed African women were most likely to be aged under 25 years, more likely to report having insufficient money to meet basic needs and less likely to be employed. Black/mixed Caribbean and ‘other ethnicity’ women were least likely to have a university education

**Table 1:** Sociodemographic characteristics of women and heterosexual men in AURAH, by ethnicity

	Women (n=666)				Heterosexual men (n=465)			
	Black/mixed African (n=325)	Black/mixed Caribbean (n=114)	White British/other (n=162)	Other ethnicity (n=65)	Black/mixed African (n=225)	Black/mixed Caribbean (n=54)	White British/other (n=146)	Other ethnicity (n=40)
Age in years (n/%)								
18–24	159 (49.7)	49 (43.8)	58 (35.8)	19 (30.7)	43 (19.5)	16 (30.2)	20 (13.8)	10 (25.6)
25–29	60 (18.8)	19 (17.0)	39 (30.3)	12 (19.4)	56 (25.3)	17 (32.1)	70 (48.3)	8 (20.5)
30–39	68 (21.3)	30 (26.8)	43 (26.5)	22 (35.5)	73 (33.0)	12 (22.6)	34 (23.5)	14 (35.9)
40+	33 (10.3)	14 (12.5)	12 (7.4)	9 (14.5)	49 (22.2)	8 (15.1)	21 (14.5)	7 (18.0)
P value				0.006				<0.001
Born in UK								
Yes	73 (23.0)	82 (75.9)	103 (64.8)	38 (59.4)	42 (18.8)	38 (71.7)	102 (69.9)	24 (61.5)
No	245 (77.0)	26 (24.1)	56 (35.2)	26 (40.6)	181 (81.2)	15 (28.3)	44 (30.1)	15 (38.5)
P value				<0.001				<0.001
Study region								
London	167 (51.4)	77 (67.5)	128 (79.0)	35 (53.9)	150 (66.7)	43 (79.6)	125 (85.6)	28 (70.0)
Outside London	158 (48.6)	37 (32.5)	34 (21.0)	30 (46.2)	75 (33.3)	11 (20.4)	21 (14.4)	12 (30.0)
P value				<0.001				<0.001
Employment status								
Employed	138 (43.1)	58 (52.7)	115 (71.0)	46 (70.8)	151 (69.3)	32 (59.3)	124 (85.5)	31 (77.5)
Student	132 (41.3)	32 (29.1)	28 (17.3)	7 (10.8)	38 (17.4)	7 (13.0)	10 (6.9)	7 (17.5)
Unemployed and other*	50 (15.6)	20 (18.2)	19 (11.2)	12 (18.5)	29 (13.3)	15 (27.8)	11 (7.6)	2 (5.0)
P value				<0.001				<0.001
Housing								
Owned/rented	226 (70.0)	81 (72.3)	124 (76.6)	44 (68.8)	170 (76.6)	31 (57.4)	128 (88.3)	26 (65.0)
Unstable and other	97 (30.0)	31 (27.7)	38 (23.5)	20 (31.3)	52 (23.4)	23 (42.6)	17 (11.7)	14 (35.0)
P value				0.45				<0.001
Money for basic needs								
Yes—sufficient money	289 (90.3)	107 (95.5)	157 (96.9)	60 (93.8)	200 (90.1)	46 (85.2)	144 (98.6)	40 (100.0)
No—not enough money	31 (9.7)	5 (4.5)	5 (3.1)	4 (6.3)	22 (9.9)	8 (14.8)	2 (1.4)	0 (0.0)
P value				0.03				<0.001†
Education—university qualification								
Yes	196 (60.3)	39 (34.2)	100 (62.1)	38 (58.5)	136 (60.4)	19 (35.2)	93 (63.7)	24 (60.0)
No	129 (39.7)	75 (65.8)	61 (37.9)	27 (41.5)	89 (39.6)	35 (64.8)	53 (36.3)	16 (40.0)
P value				<0.001				0.003
In a relationship								
Yes	224 (68.9)	74 (64.9)	104 (64.2)	56 (86.2)	163 (72.4)	33 (61.1)	80 (54.8)	22 (55.0)
No	101 (31.1)	40 (35.1)	58 (35.8)	9 (13.9)	62 (27.6)	21 (38.9)	66 (45.2)	18 (45.0)
P value				0.009				0.003

Proportions and totals may not sum to total due to rounding and missing data.

*Includes carers and retired.

†Fisher’s exact test.

AURAH, Attitudes to and Understanding of Risk of Acquisition of HIV.

Black/mixed Caribbean men were more likely to be aged under 25 years and black/mixed African and black/mixed Caribbean men were least likely to report employment and most likely to report having insufficient money to meet basic needs; black/mixed Caribbean men were least likely to have a university education.

### Sexual behaviours and history by ethnicity

Associations with ethnicity are shown in table 2 (women) and table 3 (men). There was clear evidence of an association between ethnicity and reporting of sexual risk behaviours in women. In particular, women of black/mixed African ethnicity were less likely than women of white ethnicity to report risk behaviours. In contrast, there was less evidence of variation in reported sexual behaviour among men. In both women and men, reporting an STI diagnosis in the previous year was most common among black/mixed Caribbean men and women.

**Table 2:** Sexual risk behaviours and sexual history in women, by ethnicity

	n/N	%	Unadjusted prevalence ratio	Adjusted prevalence ratio*	Adjusted prevalence ratio†
CLS with a non-regular partner/s in the last 3 months					
White British/other	61/158	38.6	1.00	1.00	1.00
Black/mixed African	81/310	26.1	0.68 (0.52–0.89)	0.67 (0.51–0.88)	0.75 (0.57–0.99)
Black/mixed Caribbean	35/111	31.5	0.82 (0.58–1.14)	0.79 (0.56–1.10)	0.86 (0.61–1.21)
Other ethnic group	13/65	20.0	0.52 (0.31–0.88)	0.67 (0.41–1.10)	0.71 (0.44–1.13)
All	190/644	29.5			
P value		0.01	0.01	0.03	0.18
Two plus CLS partners in the last 3 months					
White British/other	40/158	25.3	1.00	1.00	1.00
Black/mixed African	44/313	14.1	0.56 (0.38–0.81)	0.54 (0.37–0.80)	0.69 (0.46-1.03)
Black/mixed Caribbean	22/111	19.8	0.78 (0.49–1.24)	0.74 (0.46–1.18)	0.87 (0.54–1.39)
Other ethnic group	7/65	10.8	0.43 (0.20–0.90)	0.53 (0.25–1.13)	0.59 (0.30–1.17)
All	113/647	17.5			
P value		0.008	0.009	0.02	0.23
CLS with partner of unknown or positive HIV status in the last 3 months					
White British/other	60/158	38.0	1.00	1.00	1.00
Black/mixed African	100/313	32.0	0.84 (0.65–1.09)	0.83 (0.64–1.8)	0.93 (0.71–1.21)
Black/mixed Caribbean	35/111	31.5	0.83 (0.59–1.17)	0.81 (0.58–1.13)	0.88 (0.62–1.23)
Other ethnic group	25/65	38.5	1.01 (0.70–1.46)	1.10 (0.76–1.60)	1.18 (0.82–1.70)
All	220/647	34.0			
P value		0.46	0.45	0.24	0.49
Low self-efficacy in relation to condom use					
White British/other	39/157	24.8	1.00	1.00	1.00
Black/mixed African	60/298	20.1	0.81 (0.57–1.15)	0.75 (0.52–1.08)	0.78 (0.53–1.13)
Black/mixed Caribbean	24/106	22.6	0.91 (0.58–1.42)	0.79 (0.51–1.24)	0.81 (0.52–1.26)
Other ethnic group	17/63	27.0	1.09 (0.67–1.77)	0.94 (0.58–1.53)	0.97 (0.59–1.58)
All	140/624	22.4			
P value		0.54	0.53	0.41	0.52
Two or more new partners in the last year					
White British/other	75/158	47.5	1.00	1.00	1.00
Black/mixed African	62/298	20.8	0.44 (0.33–0.58)	0.42 (0.32–0.55)	0.51 (0.38–0.67)
Black/mixed Caribbean	22/111	19.8	0.42 (0.28–0.63)	0.44 (0.29–0.65)	0.50 (0.34–0.74)
Other ethnic group	14/62	22.6	0.48 (0.29–0.78)	0.62 (0.38–1.00)	0.67 (0.43–1.03)
All	173/629	27.5			
P value		<0.001	<0.001	<0.001	<0.001
STI diagnosed in the last year					
White British/other	29/158	18.4	1.00	1.00	1.00
Black/mixed African	58/313	18.5	1.01 (0.67–1.51)	0.98 (0.65–1.47)	0.93 (0.61–1.40)
Black/mixed Caribbean	34/111	30.6	1.67 (1.08–2.57)	1.56 (1.00–2.42)	1.50 (0.96–2.33)
Other ethnic group	13/65	20.0	1.09 (0.61–1.96)	1.25 (0.69–2.27)	1.21 (0.66–2.22)
All	134/647	20.7			
P value		0.04	0.03	0.07	0.07
Ever tested for HIV					
White British/other	125/157	79.6	1.00	1.00	1.00
Black/mixed African	253/308	82.1	1.03 (0.94–1.13)	1.10 (1.00–1.20)	1.07 (0.98–1.18)
Black/mixed Caribbean	92/107	86.0	1.08 (0.97–1.21)	1.11 (0.99–1.24)	1.10 (0.98–1.23)
Other ethnic group	49/64	76.6	0.96 (0.82–1.13)	1.00 (0.86–1.16)	0.98 (0.85–1.15)
All	519/636	81.6			
P value		0.4	0.39	0.14	0.24

*Adjusted for age, study region, education level (university degree) and relationship status.

†Adjusted for age, study region, education level (university degree), relationship status, plus alcohol and drugs.

CLS, condomless sex.

**Table 3:** Sexual risk behaviours and sexual history in heterosexual men, by ethnicity

	n/N	%	Unadjusted prevalence ratio	Adjusted prevalence ratio*	Adjusted prevalence ratio†
CLS with non-regular partner/s in the last 3 months					
White British/other	69/141	48.9	1.00	1.00	1.00
Black/mixed African	95/218	43.6	0.89 (0.71–1.11)	1.01 (0.80–1.27)	1.20 (0.93–1.55)
Black/mixed Caribbean	21/45	46.7	0.95 (0.67–1.36)	1.00 (0.71–1.42)	1.15 (0.80–1.66)
Other ethnic group	13/40	32.5	0.95 (0.41–1.07)	0.69 (0.43–1.09)	0.75 (0.48–1.16)
All	198/443	44.6			
P value		0.31	0.36	0.41	0.14
Two plus CLS partners in the last 3 months					
White British/other	49/146	33.6	1.00	1.00	1.00
Black/mixed African	68/225	30.2	0.90 (0.67–1.21)	0.97 (0.71–1.32)	1.17 (0.83–1.66)
Black/mixed Caribbean	17/54	31.5	0.94 (0.59–1.48)	0.94 (0.59–1.50)	1.08 (0.69–1.75)
Other ethnic group	8/40	20.0	0.60 (0.31–1.15)	0.61 (0.31–1.19)	0.67 (0.35–1.29)
All	142/465	30.5			
P value		0.43	0.48	0.54	0.35
CLS with partner of unknown or positive HIV status in the last 3 months					
White British/other	71/146	48.6	1.00	1.00	1.00
Black/mixed African	93/225	41.3	0.85 (0.68–1.07)	0.93 (0.73–1.18)	1.14 (0.87–1.50)
Black/mixed Caribbean	19/54	35.2	0.72 (0.49–1.08)	0.78 (0.52–1.17)	0.92 (0.62–1.38)
Other ethnic group	15/40	37.5	0.77 (0.50–1.19)	0.84 (0.54–1.30)	0.92 (0.60–1.40)
All	198/465	42.6			
P value		0.27	0.27	0.62	0.56
Low self-efficacy in relation to condom use					
White British/other	28/142	19.7	1.00	1.00	1.00
Black/mixed African	50/201	24.8	1.26 (0.84–1.90)	1.10 (0.72–1.67)	1.19 (0.75–1.88)
Black/mixed Caribbean	11/46	23.9	1.21 (0.66–2.24)	1.07 (0.60–1.91)	1.06 (0.60–1.88)
Other ethnic group	3/37	8.1	0.41 (0.13–1.28)	0.39 (0.13–1.17)	0.43 (0.14–1.29)
All	92/426	21.6			
P value		0.13	0.19	0.34	0.32
Two or more new partners in the last year					
White British/other	87/142	61.3	1.00	1.00	1.00
Black/mixed African	82/205	40.0	0.65 (0.53–0.81)	0.78 (0.63–0.96)	0.84 (0.67–1.04)
Black/mixed Caribbean	24/46	52.2	0.85 (0.63–1.16)	0.90 (0.67–1.20)	0.92 (0.68–1.25)
Other ethnic group	28/39	71.8	1.17 (0.93–1.48)	1.27 (1.03–1.56)	1.32 (1.07–1.64)
All	221/432	51.2			
P value		<0.001	<0.001	<0.001	<0.001
STI diagnosed in the last year					
White British/other	28/146	19.2	1.00	1.00	1.00
Black/mixed African	52/225	23.1	1.21 (0.80–1.82)	1.14 (0.76–1.73)	1.06 (0.69–1.63)
Black/mixed Caribbean	22/54	40.7	2.12 (1.34–3.38)	1.91 (1.20–3.04)	1.78 (1.10–2.87)
Other ethnic group	5/12.5	12.5	0.65 (0.27–1.58)	0.61 (0.25–1.47)	0.59 (0.24–1.44)
All	107/465	23.0			
P value		0.004	0.002	0.008	0.01
Ever tested for HIV					
White British/other	104/144	72.2	1.00	1.00	1.00
Black/mixed African	181/214	85.6	1.17 (1.04–1.32)	1.20 (1.06–1.35)	1.21 (1.06–1.37)
Black/mixed Caribbean	38/49	77.6	1.07 (0.90–1.29)	1.11 (0.93–1.34)	1.11 (0.92–1.34)
Other ethnic group	25/40	62.5	0.87 (0.67–1.12)	0.90 (0.71–1.16)	0.91 (0.71–1.17)
All	348/447	77.9			
P value		0.003	0.008	0.007	0.01

*Adjusted for age, study region, education level (university degree) and relationship status.

†Adjusted for age, study region, education level (university degree) and relationship status, plus alcohol and drugs.

CLS, condomless sex.

#### Heterosexual women

Black/mixed African women were approximately one-third less likely than white women to report recent condomless sex with a non-regular partner and almost half as likely to report multiple recent condomless sex partners. Similar results were seen in univariable and multivariable analyses, although there was some attenuation when alcohol and substance use were additionally adjusted for.

White women were more likely than women from any other ethnic group to report two or more new partners in the last year. In particular, women of black/mixed African and black/mixed Caribbean were more than 50% less likely to report multiple new partners in the last year compared with white women, with similar results in univariable and multivariable models.

An association between ethnicity and STI history was demonstrated. Approximately one in five women reported an STI diagnosis in the previous year and black/mixed Caribbean women were approximately 60% more likely to report a last year STI diagnosis than white women. Following additional adjustment for various high-risk behaviours, the association between ethnicity and STI history became slightly stronger (table 4).

**Table 4:** STI history by ethnicity adjusted for risk behaviours plus age, study region, education level (university degree) and relationship status, women

Risk behaviour	CLS with non-regular partner, last 3 months	CLS with two or more partners, last 3 months	Two or more new partners in the last year
STI diagnosed in the last year			
White British/other	1.00	1.00	1.00
Black/mixed African	1.01 (0.67–1.53)	1.02 (0.68–1.54)	1.04 (0.68–1.59)
Black/mixed Caribbean	1.58 (1.02–2.46)	1.61 (1.03–2.49)	1.61 (1.02–2.54)
Other ethnic group	1.28 (0.70–2.31)	1.31 (0.72–2.38)	1.22 (0.66–2.27)
All			
P value	0.08	0.08	0.09

CLS, condomless sex.

Reporting of recent condomless sex with a partner of unknown or positive HIV status, low self-efficacy for condom use and history of HIV testing were not associated with ethnicity.

#### Heterosexual men

In contrast to women, there was less evidence of an association between ethnicity and sexual behaviour in men. No association was demonstrated between ethnicity and recent condomless sex with a non-regular partner, reporting multiple recent sexual partners and reporting recent condomless sex with a partner of unknown or positive status or low self-efficacy relating to condom use. There was evidence that reporting multiple new partners in the last year was less frequent among black/mixed African men compared with white men, although this effect was lost when controlling for alcohol and drug use.

As with women, a clear association between ethnicity and STI history was demonstrated. Although black/mixed Caribbean men were not more likely than white men to report high-risk sexual behaviours, they were almost twice as likely to report an STI diagnosis in the previous year. The effect size was similar in univariable and multivariable analyses, but some attenuation was seen with the addition of alcohol and drug use to the model. The association between ethnicity and STI history was similar following additional adjustment for reported sexual behaviours (table 5).

**Table 5:** STI history by ethnicity adjusted for risk behaviours plus age, study region, education level (university degree) and relationship status, heterosexual men

Risk behaviour	CLS with non-regular partner, last 3 months	CLS with two or more partners, last 3 months	Two or more new partners in the last year
STI diagnosed in the last year			
White British/other	1.00	1.00	1.00
Black/mixed African	1.16 (0.76–1.77)	1.15 (0.76–1.73)	1.20 (0.78–1.86)
Black/mixed Caribbean	2.01 (1.25–3.24)	1.91 (1.20–3.04)	2.17 (1.34–3.50)
Other ethnic group	0.62 (0.26–1.52)	0.62 (0.25–1.50)	0.53 (0.20–1.42)
All			
P value	0.005	0.008	0.001

CLS, condomless sex.

More than three quarters of heterosexual men reported having been tested for HIV (77.9%). Black/mixed African men were about 20% more likely to report a history of HIV testing than white men.

### Sensitivity analysis

Sensitivity analyses using the alternative categorisation of ethnicity (online supplementary material) and excluding people with missing data on sexual behaviour did not materially change the results.

10.1136/sextrans-2017-053308.supp1 Supplementary file 1

## Discussion

Our results demonstrate variation in sexual risk behaviour, STI diagnosis and HIV testing history in women and heterosexual men of different ethnicities attending GUM clinics in the UK. Variation in sexual behaviour was more prominent among women than men. In particular, the prevalence of sexual risk behaviours was lower among women of black/mixed African ethnicity compared with white women. Black/mixed African women were less likely than white women to report various risk behaviours, and women of black/mixed Caribbean women were less likely than white women to report multiple new partners in the last year. Having a diagnosis of an STI in the previous 12 months was most common among women of black/mixed Caribbean ethnicity, although these women were not more likely to report high-risk sexual behaviours than white women. The association between STI history and black Caribbean ethnicity was not attenuated by adjustment for risk behaviours. Black/mixed African men were less likely than white men to report two or more new partners in the last year and were more likely to have tested for HIV. There was no significant difference in the proportion of men of different ethnicities reporting recent condomless sex with a non-regular partner/s or multiple condomless sex partners. However, black/mixed Caribbean men were much more likely to have been diagnosed with an STI in the previous year than white men. This association was not attenuated by adjustment for risk behaviours.

Direct comparison with other UK studies is challenging due to differences in populations and the risk behaviours included in our questionnaire. Our findings contrast with some UK studies, including Natsal-2, which found that sexual risk behaviours were higher in black African and Caribbean women and/or men than in individuals from other ethnic groups.^2 8^ There are significant differences between the study populations of Natsal-2 and AURAH which may explain this, the most important of which is that the Natsal study populations are nationally representative while AURAH recruited from GUM clinics. A higher prevalence of sexual risk behaviour would be expected in GUM settings, as demonstrated in the LIVITY study, a study of sexual behaviour reported by Black Caribbean GUM clinic attendees in London.^9^ In addition, considerable time has passed between Natsal-2 and AURAH, and there are differences in the prevalence of some sexual behaviour measures reported between Natsal-2 (1999–2000) and Natsal-3 (2010–2012).^17 18^ For example, the proportion of male and female Natsal-3 participants reporting recent vaginal sex was significantly lower in Natsal-3, as was the proportion of men reporting at least one new female partner in the previous year.^19^ In contrast, the number of male lifetime sexual partners reported by women increased.^19^

In our study, prevalence of recent history of STI did not follow the same pattern across ethnicity groups as reported sexual risk behaviour. This suggests that markers such as condomless sex and number of sexual partners may be insufficient in characterising STI risk. This is consistent with previous studies demonstrating the importance of factors such as the partner selection in determining STI risk.^19^ Moreover, we demonstrate that this pattern of disproportionate STI experience among people of black Caribbean ethnicity, apparently unexplained by individual risk behaviour, remains more than a decade after its initial observation. There is evidence from other studies that ethnic disparities in STI and HIV are not fully explained by individual behaviour: findings from the UK, USA and Canada suggest disparities in sexual health outcomes in black ethnicity individuals persist even when controlling for behaviour.^8 20–22^ Using data from the National Longitudinal Study of Adolescent to Adult Health (Add Health), Hallfors *et al* investigated the relationship of sexual behaviour and substance use with risk of STI diagnosis in a nationally representative sample of young people in the USA.^22^ Participants were stratified into risk groups based on reported sexual behaviour and substance use, and the prevalence of STI was compared between white and black ethnicity individuals in each strata. Hallfors *et al* report that even in the lowest risk group, the odds of new STI diagnosis in black ethnicity individuals was more than seven times that of white individuals.^22^ Similarly, Fenton *et al* demonstrated that although female black Caribbean participants in Natsal-2 were more likely to report a positive STI history, there was no evidence of significant differences in the proportion of women of different ethnicities reporting partner concurrency or a recent new partner.^8^

Sexual network characteristics are associated with STI risk.^23–26^ Network characteristics associated with STI transmission include a high number of partner changes and high baseline STI prevalence.^22 24^ Partner concurrency has been highlighted in mathematical models as a risk for STI and HIV transmission,^27^ and may influence STI risk within sexual networks.^28^ Individual risk is also influenced by partner behaviour and network/s.^24^ Our finding of a disparity between STI history and reported sexual risk behaviour suggests that STI risk is not determined exclusively at the individual level but is affected by interactions within sexual networks.

A key strength of this study is its focus on sexual behaviour and this study adds to previous work in this area.^2 7 9^ Additionally, the number of black/mixed African and black/mixed Caribbean participants recruited allows reasonably well- powered comparisons between these groups. There are a number of limitations. Social desirability bias may impact on the reliability of the responses elicited by our questionnaire. However, results show reporting of STI history consistent with local epidemiology, so it is unlikely that bias alone explains our findings, as it is unclear why this would pertain to self-reported behaviour measures but not to self-reported STI history. Although the number of participants recruited to AURAH permitted analysis between black ethnic groups, these remain broad categories and it is probable that intragroup variation remains. Additionally, while separate questionnaires were produced for men and women, with specific questions for MSM, the use of one questionnaire to evaluate risk in both heterosexual and homosexual/bisexual groups may be a limitation. For example, a number of questions focused on risk factors associated with STI and HIV in MSM (eg, chemsex), which may be less relevant for heterosexual individuals. Furthermore, our questionnaire did not cover a number of behaviours which have been linked to STI/HIV in heterosexuals including partner concurrency,^27 28^ partner risk behaviours^23 26^ and assortative sexual partner selection.^19 20 22^ This is an important limitation, and future research may benefit from a greater focus on individual risk groups rather than attempting broader analysis of behaviour. Finally, our study was recruited from a GUM population whose demographics and sexual behaviours are likely to be different from the general population; this limits the generalisability of our findings beyond this setting.

## Conclusion

Our findings are consistent with national epidemiology^1^ in demonstrating a higher prevalence of STI history in individuals of black/mixed Caribbean ethnicity compared with white ethnicity individuals; however, this finding is not associated with higher reporting of sexual risk behaviours. STI risk is likely to be related to both individual and population factors, including STI prevalence in one’s sexual network. It is critical that research in this area seeks to understand the breadth of determinants of sexual health and does not stigmatise ethnic groups who have a disproportionate prevalence of STI disease.

**Key messages**

- STIs in the UK are unevenly distributed. Among heterosexual men and women, individuals of black Caribbean ethnicity are disproportionately affected by STIs.
- In our questionnaire-based study, a history of STI was reported more frequently by men and women of black/mixed Caribbean ethnicity, compared with white men and women. This was not explained by reported sexual behaviours.
- Sexual history and outcomes are likely to be influenced by factors beyond the individual, including partner behaviour and sexual networks.
