# Supplementary material for: Ethnicity and sexual risk in heterosexual people attending sexual health clinics in England: a cross-sectional, self-administered questionnaire study
Source: Sex Transm Infect. 2018 Mar 8;94(5):384–91. doi: 10.1136/sextrans-2017-053308 (PMC6204969; doi:10.1136/sextrans-2017-053308)
Supplement: Supplementary file 1 [file sextrans-2017-053308supp001.docx]

The ethnic groups from which participants self-selected their ethnicity is listed below.

1. White
   1. White British
   2. White Irish
   3. White other
2. Black or Black British
   1. Black African
   2. Black Caribbean
   3. Black other
3. Asian or Asian British
   1. Indian
   2. Pakistani
   3. Bangladeshi
   4. Asian other
4. Mixed
   1. White and Black African
   2. White and Black Caribbean
   3. White and Asian
   4. Mixed other
5. Chinese or other ethnic group
   1. Chinese
   2. Any other ethnic group

Supplementary Table 1 Self-reported ethnicity of heterosexual AURAH participants, with original ethnicity classification – (i) White ethnicity, (ii) Black/mixed African, (iii)Black/mixed Caribbean, (iv) all other ethnic groups.

|  | Gender (n/%) | |  |
| --- | --- | --- | --- |
| Ethnic group | Male | Female | Total (n/%) |
| White ethnicity | 146 (31.4) | 162 (24.3) | 308 (27.2) |
| Black/mixed African | 225 (48.4) | 325 (48.8) | 550 (48.6) |
| Black/mixed Caribbean | 54 (11.6) | 114 (17.1) | 168 (14.9) |
| All other ethnic groups | 40 (8.6) | 65 (9.8) | 105 (9.3) |
| Total | 465 | 666 | 1,131 |

Supplementary Table 2 Self-reported ethnicity of heterosexual AURAH participants revised, ethnicity classification – (i) White ethnicity, (ii) Black African, (iii)Black Caribbean, (iv) all other ethnic groups.

|  | Gender (n/%) | |  |
| --- | --- | --- | --- |
| Ethnic group | Male | Female | Total (n/%) |
| White ethnicity | 146 (31.4) | 162 (24.3) | 308 (27.2) |
| Black African | 213 (45.8) | 306 (46.0) | 519 (45.9) |
| Black Caribbean | 48 (10.3) | 92 (13.8) | 140 (12.4) |
| All other ethnic groups | 58 (12.5) | 106 (15.9) | 164 (14.5) |
| Total | 465 | 666 | 1,131 |

Supplementary table 3 Sensitivity analysis with women - ethnic groups = White, Black African, Black Caribbean, Other ethnicity

|  | Women | |  |  |  |
| --- | --- | --- | --- | --- | --- |
|  | n/N | % | Unadjusted Prevalence Ratio | Adjusted Prevalence Ratio^a^ | Adjusted Prevalence Ratio^b^ |
|  |  |  |  |  |  |
| CLS with a non-regular partner/s last 3 months | | | |  |  |
| White British/Other | 61/158 | 38.6 | 1 | 1.00 | 1.00 |
| Black African | 74/292 | 25.3 | 0.66 (0.50-0.87) | 0.65 (0.49-0.86) | 0.73 (0.56-0.97) |
| Black Caribbean | 27/90 | 30.0 | 0.78 (0.54-1.13) | 0.78 (0.54-1.19) | 0.86 (0.59-1.25) |
| Other ethnicities | 28/104 | 26.9 | 0.70 (0.48-1.01) | 0.77 (0.53-1.10) | 0.80 (0.56-1.13) |
| All | 190/644 | 29.5 |  |  |  |
| *p value* |  | 0.03 | 0.02 | 0.02 | 0.19 |
| 2+ CLS partners last 3 months | | | |  |  |
| White British/Other | 40/158 | 25.3 | 1.00 | 1.00 | 1.00 |
| Black African | 38/295 | 12.9 | 0.51 (0.34-0.76) | 0.50 (0.34-0.74) | 0.64 (0.42-0.98) |
| Black Caribbean | 17/90 | 18.9 | 0.75 (0.45-1.24) | 0.74 (0.45-1.23) | 0.92 (0.55-1.55) |
| Other ethnicities | 18/104 | 17.3 | 0.68 (0.42-1.13) | 0.73 (0.44-1.20) | 0.76 (0.48-1.19) |
| All | 113/647 |  |  |  |  |
| *p value* |  | 0.01 | 0.01 | 0.01 | 0.18 |
| CLS with partner of unknown or positive HIV status in last 3 months | | | | |  |
| White British/Other | 60/158 | 38.0 | 1.00 | 1.00 | 1.00 |
| Black African | 94/295 | 31.9 | 0.84 (0.65-1.09) | 0.83 (0.64-1.08) | 0.93 (0.71-1.23) |
| Black Caribbean | 30/90 | 33.3 | 0.88 (0.62-1.25) | 0.88 (0.63-1.25) | 0.98 (0.69-1.29) |
| Other ethnicities | 36/104 | 34.6 | 0.91 (0.65-1.27) | 0.92 (0.66-1.29) | 0.96 (0.69-1.33) |
| All | 220/647 | 34.0 |  |  |  |
| *p value* |  | 0.63 | 0.62 | 0.56 | 0.97 |
| Low self-efficacy in relation to condom use | | | |  |  |
| White British/Other | 39/157 | 24.8 | 1.00 | 1.00 | 1.00 |
| Black African | 55/281 | 19.0 | 0.79 (0.55-1.13) | 0.73 (0.50-1.06) | 0.76 (0.52-1.11) |
| Black Caribbean | 17/85 | 20.0 | 0.81 (0.49-1.33) | 0.70 (0.43-1.16) | 0.71 (0.43-1.18) |
| Other ethnicities | 29/101 | 28.7 | 1.16 (0.77-1.74) | 1.00 (0.66-1.51) | 1.02 (0.67-1.53) |
| All | 140/624 | 22.4 |  |  |  |
| *p value* |  | 0.22 | 0.21 | 0.19 | 0.26 |
| 2 or more new partners in last year | | | |  |  |
| White British/Other | 75/158 | 47.5 | 1.00 | 1.00 | 1.00 |
| Black African | 56/280 | 20.0 | 0.42 (0.32-0.56) | 0.40 (0.31-0.53) | 0.49 (0.37-0.65) |
| Black Caribbean | 16/90 | 17.8 | 0.37 (0.23-0.60) | 0.42 (0.26-0.66) | 0.49 (0.31-0.76) |
| Other ethnicities | 26/101 | 25.7 | 0.54 (0.37-0.79) | 0.61 (0.42-0.88) | 0.65 (0.46-0.91)** |
| All | 173/629 | 27.5 |  |  |  |
| *p value* |  | <0.001 | <0.001 | <0.001 | <0.001 |
| STI diagnosed in last year | | | |  |  |
| White British/Other | 29/158 | 18.4 | 1.00 | 1 | 1.00 |
| Black African | 54/295 | 18.3 | 1.00 (0.66-1.50) | 0.96 (0.64-1.45) | 0.91 (0.59-1.38) |
| Black Caribbean | 30/90 | 33.3 | 1.81 (1.17-2.82) | 1.74 (1.11-2.72) | 1.65 (1.05-2.60) |
| Other ethnicities | 21/104 | 20.2 | 1.10 (0.66-1.82) | 1.14 (0.69-1.90) | 1.13 (0.68-1.88) |
| All | 134/647 | 20.7 |  |  |  |
| *p value* |  | 0.02 | 0.01 | 0.03 | 0.02 |
| Ever tested for HIV | | | |  |  |
| White British/Other | 125/157 | 79.6 | 1.00 | 1.00 | 1.00 |
| Black African | 239/290 | 82.4 | 1.04 (0.94-1.14) | 1.10 (1.00-1.21) | 1.08 (0.98-1.18) |
| Black Caribbean | 76/86 | 88.4 | 1.11 (0.99-1.24) | 1.13 (1.01-1.26) | 1.11 (0.99-1.24) |
| Other ethnicities | 79/103 | 76.7 | 0.96 (0.84-1.10) | 1.01 (0.89-1.15) | 1.01 (0.88-1.15) |
| All | 519/636 | 81.6 |  |  |  |
| *p value* |  | 0.19 | 0.13 | 0.08 | 0.20 |
| CLS – condomless sex  ^a^ Adjusted for age, study region, education level (university degree) and relationship status | | | | |  |
| ^b^ Adjusted as per ^a^ plus alcohol and drugs | | |  |  |  |
|  | | |  |  |  |

Supplementary table 4 Sensitivity analysis men with- ethnic groups = White, Black African, Black Caribbean, Other

|  | Heterosexual men | |  |  |  |
| --- | --- | --- | --- | --- | --- |
|  | n/N | % | Unadjusted Prevalence Ratio | Adjusted Prevalence Ratio^a^ | Adjusted Prevalence Ratio^b^ |
|  |  |  |  |  |  |
| CLS with a non-regular partner/s last 3 months | | | |  |  |
| White British/Other | 69/141 | 48.9 | 1 | 1 | 1 |
| Black African | 90/208 | 43.3 | 0.88 (0.70-1.11) | 1.00 (0.79-1.27) | 1.19 (0.92-1.54) |
| Black Caribbean | 18/40 | 45 | 0.92 (0.63-1.35) | 0.98 (0.67-1.43) | 1.11 (0.75-1.63) |
| Other ethnicities | 21/55 | 38.2 | 0.78 (0.54-1.14) | 0.82 (0.57-1.17) | 0.90 (0.64-1.28) |
| All | 198/444 | 44.6 |  |  |  |
| *p value* |  | *0.54* | *0.55* | *0.7* | *0.37* |
| 2+ CLS partners last 3 months | | | |  |  |
| White British/Other | 49/146 | 33.6 | 1 | 1 | 1 |
| Black African | 68/213 | 31.9 | 0.95 (0.70-1.29) | 1.03 (0.75-1.40) | 1.27 (0.89-1.80) |
| Black Caribbean | 16/48 | 33.3 | 0.99 (0.63-1.57) | 1.01 (0.63-1.61) | 1.16 (0.72-1.89) |
| Other ethnicities | Sep-58 | 15.5 | 0.46 (0.24-0.88) | 0.48 (0.25-0.92) | 0.54 (0.28-1.02) |
| All | 142/465 | 30.5 |  |  |  |
| *p value* |  | *0.07* | *0.13* | *0.13* | *0.05* |
| CLS with partner of unknown or positive HIV status in last 3 months | | | | |  |
| White British/Other | 71/146 | 48.6 | 1 | 1 | 1 |
| Black African | 89/213 | 41.8 | 0.86 (0.68-1.08) | 0.93 (0.73-1.19) | 1.16 (0.87-1.53) |
| Black Caribbean | 16/48 | 33.3 | 0.69 (0.44-1.06) | 0.75 (0.48-1.16) | 0.88 (0.58-1.36) |
| Other ethnicities | 22/58 | 37.9 | 0.78 (0.54-1.13) | 0.85 (0.59-1.23) | 0.95 (0.66-1.37) |
| All | 198/465 | 42.6 |  |  |  |
| *p value* |  | *0.22* | *0.23* | *0.56* | *0.5* |
| Low self-efficacy in relation to condom use | | | |  |  |
| White British/Other | 28/142 | 19.7 | 1 | 1 | 1 |
| Black African | 49/191 | 26.7 | 1.30 (0.86-1.96) | 1.14 (0.75-1.73) | 1.24 (0.78-1.97) |
| Black Caribbean | Oct-42 | 23.8 | 1.21 (0.64-2.28) | 1.03 (0.57-1.85) | 1.02 (0.58-1.81) |
| Other ethnicities | May-51 | 9.8 | 0.50 (0.20-1.22) | 0.49 (0.20-1.18) | 0.53 (0.21-1.29) |
| All | 92/426 | 21.6 |  |  |  |
| *p value* |  | *0.09* | *0.13* | *0.3* | *0.27* |
| 2 or more new partners in last year | | | |  |  |
| White British/Other | 87/142 | 61.3 | 1 | 1 | 1 |
| Black African | 80/195 | 41 | 0.67 (0.54-0.83) | 0.80 (0.65-0.98) | 0.86 (0.69-1.08) |
| Black Caribbean | 23/42 | 54.8 | 0.89 (0.66-1.21) | 0.97 (0.73-1.30) | 1.00 (0.75-1.35) |
| Other ethnicities | 31/53 | 58.5 | 0.95 (0.73-1.24) | 1.03 (0.82-1.31) | 1.08 (0.85-1.38) |
| All | 221/432 | 51.2 |  |  |  |
| *p value* |  | *0.002* | *0.003* | *0.11* | *0.31* |
| STI diagnosed in last year | | | |  |  |
| White British/Other | 28/146 | 19.2 | 1 | 1 | 1 |
| Black African | 50/213 | 23.5 | 1.22 (0.81-1.85) | 1.15 (0.76-1.76) | 1.08 (0.70-1.66) |
| Black Caribbean | 20/48 | 41.7 | 2.17 (1.35-3.49) | 1.95 (1.21-3.13) | 1.81 (1.11-2.95) |
| Other ethnicities | 9/58 | 15.5 | 0.81 (0.41-1.61) | 0.78 (0.39-1.56) | 0.75 (0.47-1.51) |
| All | 107/465 | 23 |  |  |  |
| *p value* |  | *0.06* | *0.003* | *0.01* | *0.02* |
| Ever tested for HIV | | | |  |  |
| White British/Other | 104/144 | 72.2 | 1 | 1 | 1 |
| Black African | 173/202 | 85.6 | 1.19 (1.06-1.33) | 1.22 (1.08-1.37) | 1.23 (1.08-1.40) |
| Black Caribbean | 34/43 | 79.1 | 1.09 (0.91-1.32) | 1.13 (0.93-1.38) | 1.13 (0.93-1.39) |
| Other ethnicities | 37/58 | 63.8 | 0.88 (0.71-1.10) | 0.90 (0.73-1.11) | 0.91 (0.74-1.13) |
| All | 348/447 | 77.9 |  |  |  |
| *p value* |  | *0.001* | *0.002* | *0.001* | *0.002* |
| CLS – condomless sex | | | | | |
| ^a^ Adjusted for age, study region, education level (university degree) and relationship status | | | | | |
| ^b^ Adjusted as per ^a^ plus alcohol and drugs | | |  |  |  |
|  | | |  |  |  |
